# Production of three phenylethanoids, tyrosol, hydroxytyrosol, and salidroside, using plant genes expressing in *Escherichia coli*

**DOI:** 10.1038/s41598-017-02042-2

**Published:** 2017-05-31

**Authors:** Daeun Chung, So Yeon Kim, Joong-Hoon Ahn

**Affiliations:** Department of Integrative Bioscience and Biotechnology, Bio/Molecular Informatics Center, Konkuk University, Seoul, 05029 Republic of Korea

## Abstract

Polyphenols, which include phenolic acids, flavonoids, stilbenes, and phenylethanoids, are generally known as useful antioxidants. Tyrosol, hydroxytyrosol, and salidroside are typical phenylethanoids. Phenylethanoids are found in plants such as olive, green tea, and *Rhodiola* and have various biological activities, including the prevention of cardiovascular diseases, cancer, and brain damage. We used *Escherichia coli* to synthesize three phenylethanoids, tyrosol, hydroxytyrosol, and salidroside. To synthesize tyrosol, the aromatic aldehyde synthase (*AAS*) was expressed in *E. coli*. Hydroxytyrosol was synthesized using *E. coli* harboring *AAS* and *HpaBC*, which encodes hydroxylase. In order to synthesize salidroside, 12 uridine diphosphate-dependent glycosyltransferases (UGTs) were screened and UGT85A1 was found to convert tyrosol to salidroside. Using *E. coli* harboring *AAS* and UGT85A1, salidroside was synthesized. Through the optimization of these three *E. coli* strains, we were able to synthesize 531 mg/L tyrosol, 208 mg/L hydroxytyrosol, and 288 mg/L salidroside, respectively.

## Introduction

Many countries have traditional foods or medicines; olive oil in southern Europe and the Middle East and green tea from Asia are considered regional traditional foods. Olive oil and green tea contain many antioxidants due to presence of phenolic compounds. Antioxidant protects cells and tissues from oxidative injury^1^, which can cause Parkinson’s disease, Alzheimer’s disease, other forms of dementia, cancer, and heart disease. Historically, olive oil has been called a “miracle food” because it aids in digestion and combats skin cancer. Also, olive oil has been shown to effect on cardiovascular disease and certain types of cancers^2^. Two major ingredients in olive are tyrosol and hydroxytyrosol, both of which are considered bioactive ingredients having various biological activities. It has been proven that tyrosol can lower the risk of developing Alzheimer’s disease^3^. Salidroside, a glucoside of tyrosol, is one of the major ingredients of the medicinal herb, *Rhodiola* ^4^. Salidroside exhibits various biological activities, including nerve and brain cell protection, bone loss reduction and weight reduction^5–8^. Tyrosol, hydroxytyrosol, and salidroside belong to a group of plant phenolic compounds called phenylethanoids.

**Figure 1:**
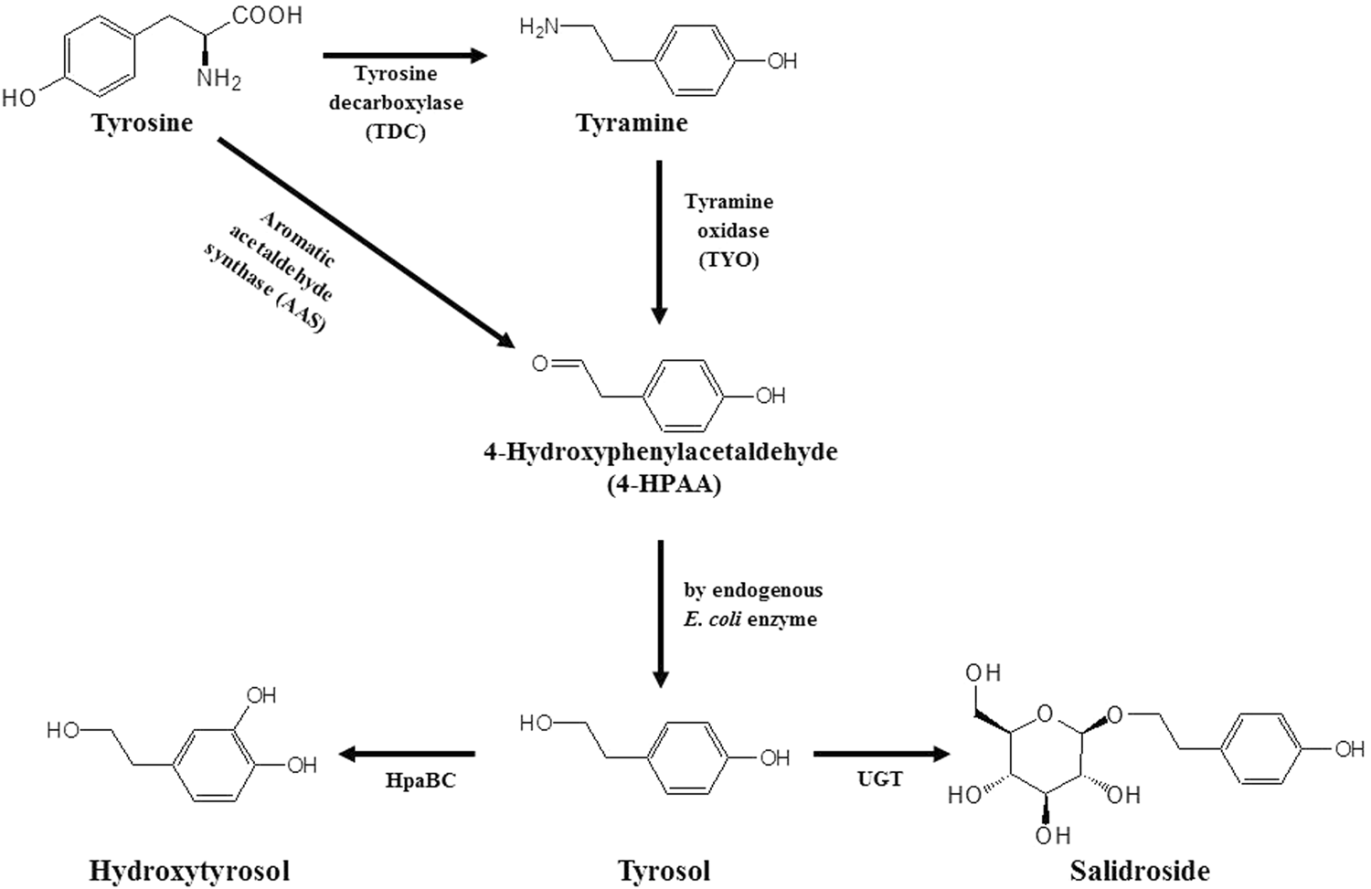
Scheme of biosynthesis of three phenylethanoids, tyrosol, hydroxytyrosol, and salidroside.

Tyrosol is synthesized from tyrosine. There are two possible biosynthesis pathways for tyrosol synthesis in plants. In the first proposed pathway, tyrosine is converted into tyramine by tyrosine decarboxylase (TDC). Subsequent oxidation and reduction of tyramine result in the formation of tyrosol^9^ (Fig. 1). However, growing evidence indicates that tyrosol is synthesized via tyramine, as TDC was identified in *Rhodiola sachalinensis* ^10, 11^. The carbon backbone of tyrosol is C6-C2. Plant phenolic compounds have been synthesized via cinnamic acid, which has a C6-C3 carbon backbone and is derived from phenylalanine. In order to synthesize other phenolic compounds such as flavonoids and stilbenes, malonyl-CoA serves as a carbon donor to transfer two carbons to hydroxycinnamic acid. The synthesis of C6-C1 phenolic compounds, such as benzoic acid relies on the coenzyme A-dependent β-oxidation of cinnamoyl-CoA^11^. These results support that tyrosol is synthesized through tyrosine decarboxylation.

Salidroside is synthesized from tyrosol by a uridine diphosphate dependent glycosyltransferase (UGT). UGT73B6 from *R. sachalinensis* was found to be involved in the biosynthesis of salidroside although it also produces icariside D2 using tyrosol^12, 13^.

*Escherichia coli* has been widely used to synthesize diverse phytochemicals. Biological synthesis using microorganisms has advantages over enzymatic and chemical syntheses; it does not require expensive cofactors and it confers regioselectivity and stereoselectivity. Tyrosol, hydroxytyrosol, and salidroside have been previously synthesized in *E. coli*. There are two possible routes for the production of tyrosol in *E. coli*. In the first method using tyrosine as a substrate, *TDC* and *TYO* were introduced into an *E. coli feaB* (phenylacetaldehyde dehydrogenase) deletion strain^14^. FeaB competes for 4-hydroxyphenylacetaldehyde (4-HPAA) and produces 4-hydroxyphenylacetate instead of tyrosol. Therefore, deletion of *feaB* results in and increase in tyrosol production by approximately 43%. Using this *E. coli* strain, Satoh *et al*.^14^ were able to synthesize 69 mg/L tyrosol. Second route of tyrosol production in *E. coli* utilized *aro10* from yeast encoding pyruvate decarboxylase that converts 4-hydroxyphenylpyruvate to 4-HPAA. Bai *et al*.^13^ used *aro10* and other genes for the biosynthesis of tyrosine, in addition to several *E. coli* mutants to produce tyrosol. This group also introduced UGT into the tyrosine producing *E. coli* strain to synthesize salidroside^13^. Hydroxytyrosol was synthesized using tyrosine hydroxylase (TH), L-DOPA decarboxylase (DDC) and tyramine oxidase (TYO)^14^.

Studies of plant aromatic amino acid decarboxylases (AAADs) revealed that some AAADs are bifunctional enzymes capable of catalyzing both decarboxylation and oxidation^15–17^. This family of AAADs is known as aromatic aldehyde synthases (AASs). AAS converts tyrosine into 4-HPAA, and then 4-HPAA can be converted into tyrosol in *E. coli*. In this report, we used plant AAS to synthesize tyrosol, hydroxytyrosol, and salidroside in *E. coli*. Through the metabolic engineering of *E. coli*, 531 mg/L tyrosol and 208 mg/L hydroxytyrosol were synthesized. Furthermore, in order to synthesize salidroside, we screened 12 UGTs from *Arabidopsis thaliana*. Using engineered *E. coli* harboring *AAS* and the identified UGT, we could synthesize 288 mg/L salidroside.

## Results

### Synthesis of tyrosol using AAS in *E. coli*

**Figure 2:**
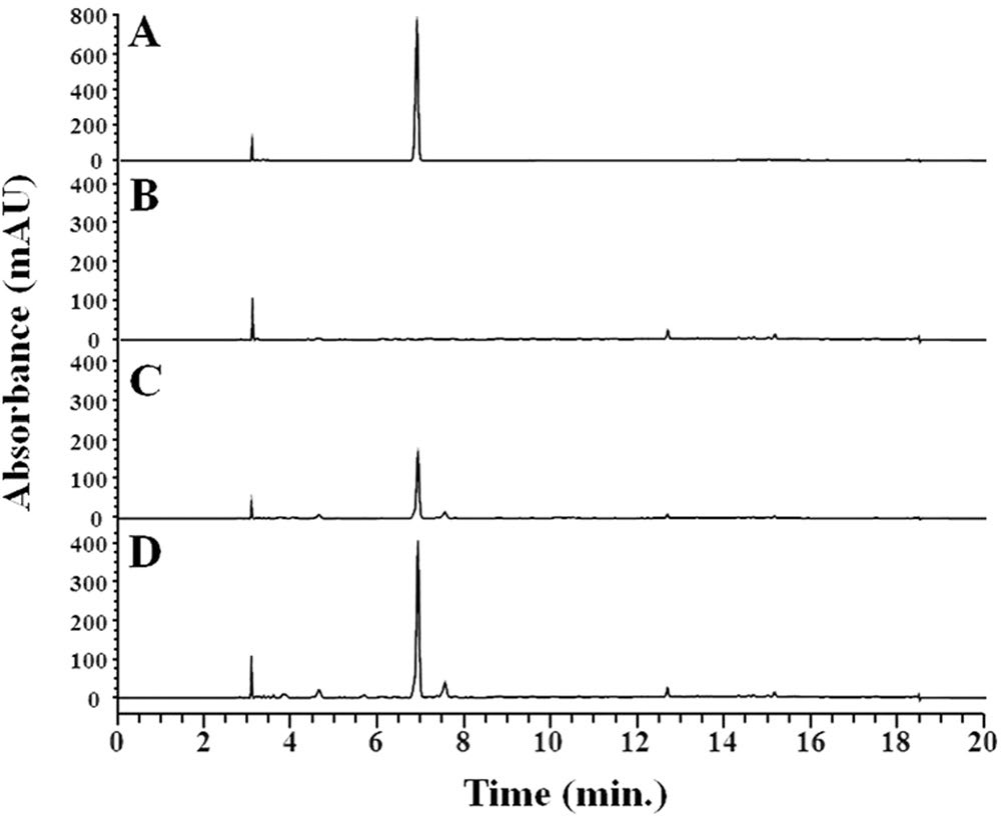
Production of tyrosol using *E. coli* harboring AAS. (**A**) standard tyrosol; (**B**) AAS from *Arabidopsis thaliana*; (**C**) AAS from *Petunia hybrid*; (**D**) AAS from *Petroselinum crispum*.

For the synthesis of tyrosol in *E. coli*, we constructed the pathway from tyrosine to tyrosol using AAS. AAS is a bifunctional enzyme that converts tyrosine into 4-HPAA oxidation^15–17^. In *E. coli*, 4-HPAA is converted to tyrosol by alcohol dehydrogenase(s)^14^. We tested three different *AAS* genes from *A. thaliana*, *Petunia hybrid*, and *Petroselinum crispum*. After induction of each protein, tyrosine (100 μM) was added to the culture. The culture filtrates from the *E. coli* strains harboring each *AAS* were analyzed using HPLC. *E. coli* harboring *AAS* from *P. crispum* produced approximately 12.6 mg/L tyrosol (Fig. 2). *E. coli* harboring *AAS* from *A. thaliana* did not produce tyrosol and *E. coli* harboring *AAS* from *P. hybrid* produced approximately 5.3 mg/L tyrosol. The structure of the reaction product in Fig. 1 was determined to be tyrosol using NMR. We decided to use the PcAAS for further experiments. We could not observe the reaction intermediates such as 4-HPAA, suggesting that AAS and the endogenous *E. coli* reductase, which converts 4-HPAA into tyrosol, were balanced in the production of tyrosol.

**Figure 3:**
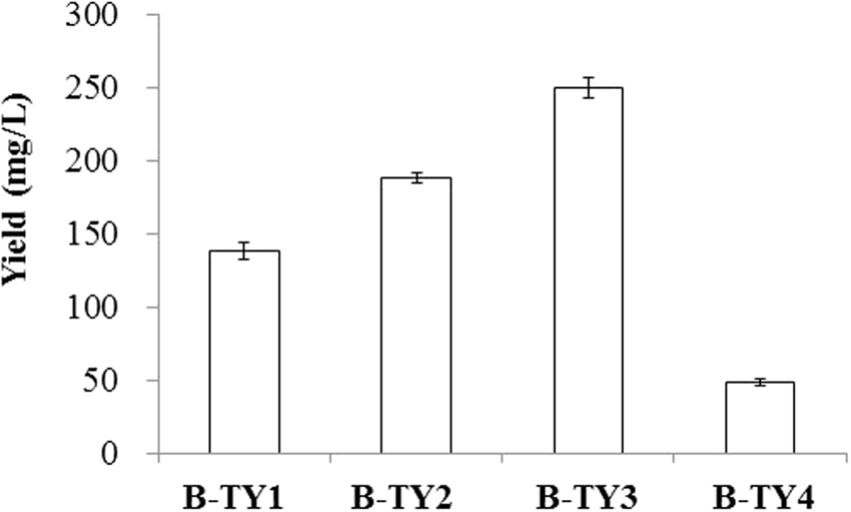
Effect of different *E. coli* strain on the production of tyrosol. B-TY1, wild type harboring *AAS*; B-TY2, *tyrR* and *pheA* deletion mutant harboring AAS; B-TY3, *tyrR*, *pheA*, and *feaB* deletion mutant harboring AAS; B-TY4, wild type harboring TDC and TYO.

Tyrosol is synthesized from tyrosine. Therefore, it is likely that total tyrosine content is correlated with the final tyrosol yield. The reaction intermediate, the 4-HPAA, can be converted into 4-hydroxyphenylacetic acid by phenylacetaldehyde dehydrogenase (feaB), which competes with the *E. coli* alcohol dehydrogenase for 4-HPAA and results in reduction of the final yield of tyrosol. We used two mutants B-TP and B-TPF (Table 1). The strain B-TP produced more tyrosine than did the wild type due to the deletions of the transcriptional regulator, *tyrR*, which is inhibited by tyrosine, and *pheA* encoding a chorismate mutase/prephenate dehydratase that drives prephenate toward the biosynthesis of the phenylalanine instead of biosynthesis of tyrosine^18, 19^. The strain B-TPF contains deletions in *feaB*, *tyrR*, and *pheA*. FeaB encodes phenylacetaldehyde dehydrogenase, which converts 4-HPAA to 4-hydroxyphenylacetate (4-HPA). Deletion of this gene was expected to increase more 4-HPAA for tyrosol synthesis. As an alternative route for the tyrosol biosynthesis, we used tyrosine decarboxylase (*TDC*) from *Papaver somniferum* and tyrosine oxidase (*TYO*) from *Micrococcus luteus*. These two gene was transformed into *E. coli* BL21(DE3). Strain B-TY1 produced 138.9 mg/L of tyrosol, B-TY2 produced 188.1 mg/L, and B-TY3 produced 250.4 mg/L (Fig. 3). This result demonstrated that the contents of tyrosine and 4-HPAA are critical to the final yield of tyrosol. However, B-TY4, which had TDC and TYO, produced only 49.2 mg/L tyrosol.

**Table 1:** Plasmids, *Escherichia coli* strains, and primers used in this study.

Plasmids or *E. coli* strain or Primers	Relevant properties or genetic marker		Source or reference
**Plasmids**			
pACYCDDuet		P15A ori, Cm^r^	Novagen
pCDFDuet		CloDE13 ori, Str^r^	Novagen
pGEX		f1 ori, Amp^r^	GE Healthcare
**Constructs**			
pC-PcAAS		pCDFDuet carrying aromatic aldehyde synthase (AAS) from *Petroselinum crispum*	This study
pC-PcAAS-HpaBC		pCDFDuet carrying aromatic aldehyde synthase (AAS) from and *P. crispum* and *HpaBC* from *Escherichia coli*	This study
pE-HpaBC		pETDuet carrying *HpaBC* from *E. coli*	This study
pC-TDC-TYO		pCDFDuet carrying TDC from *Papaver somniferum* and TYO from *Micrococcus luteus*	This study
**Strains**			
BL21 (DE3)		F^−^ *ompT hsdS*_*B*_(r_B_^−^ m_B_^−^) *gal dcm lon* (DE3)	Novagen
B-TP		BL21(DE3) *ΔtyrR*::*FRT- ΔPheA::FRT-kan* ^*R*^ *-FRT*	36
B-TPF		BL21(DE3) *ΔtyrR*::*FRT- ΔPheA:: ΔfeaB-FRT FRT-kan* ^*R*^ *-FRT*	This study
B-TY1		BL21 (DE3) harboring pC-PcAAS	This study
B-TY2		B-TP harboring pC-PcAAS	This study
B-TY3		B-TPF harboring pC-PcAAS	This study
B-TY4		BL21 (DE3) harboring pC-TDC-TYO	This study
B-SAL1		BL21 (DE3) harboring pC-PcAAS and pG-AtUGT85A1	This study
B-SAL2		B-TPF harboring pC-PcAAS and pG-AtUGT85A1	This study
B-HTY1		BL21 (DE3) harboring pC-PcAAS-HpaBC	This study
B-HTY2		BL21 (DE3) harboring pC-PcAAS and pE-HpaBC	This study
B-HTY3		B-TPF harboring pC-PcAAS-HpaBC	This study

**Figure 4:**
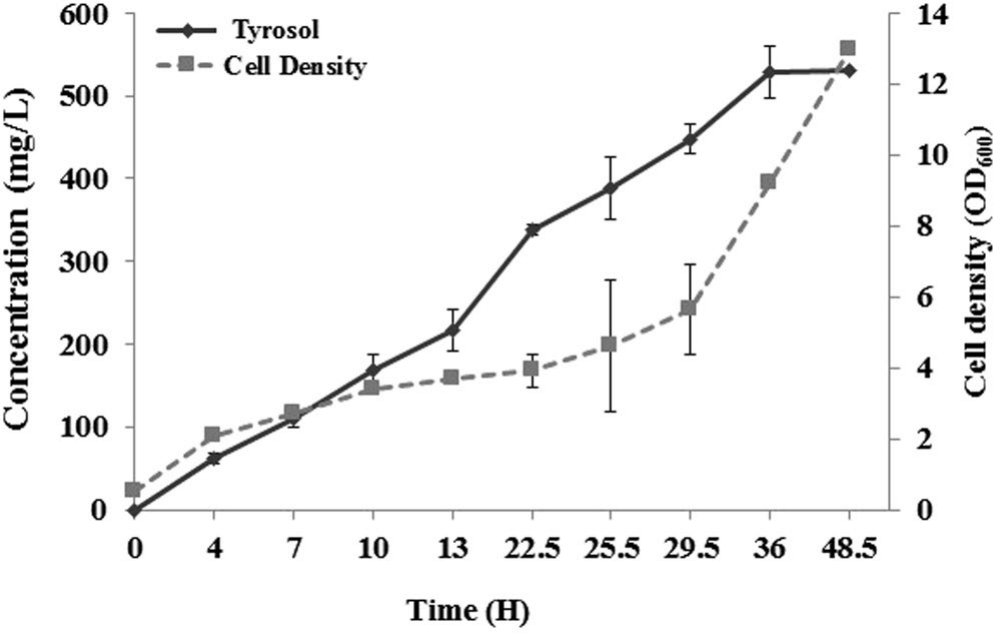
Production of tyrosol using strain B-TY3 after optimization of incubation time and cell concentration.

Strain B-TY3 was used to synthesize tyrosol. The optimal culturing temperature and the initial cell concentration were determined. Tyrosol production at 30 °C in B-TY3 was better than that at 25 °C or 37 °C. The production at 25 °C and 37 °C was 57.7% and 20.4% of that at 30 °C, respectively. The initial cell concentration was tested at OD_600_ = 0.5, 1, 1.5, 2. 2.5, and 3. The production tyrosol at OD_600_ = 0.5 was highest, and production declined with increasing cell concentrations. Using the optimized incubation time and cell concentration of B-TY3, the production of tyrosol was monitored for 48 h. The production of tyrosol continued to increase until 36 h, at which 539.4 mg/L tyrosol was produced (Fig. 4). After 36 h, tyrosol production did not increase.

### Synthesis of salidroside in *E. coli*

We attempted to synthesize salidroside in *E. coli*. Salidroside is 2-(4-hydroxyphenyl) ethyl β-D-glucopyranoside. In order to synthesize salidroside from tyrosol, a uridine-dependent glycosyltransferase (UGT), which transfers glucose from UDP-glucose to an acceptor molecule such as tyrosol, was needed. We screened 12 UGTs from *A. thaliana* to identify a UGT that synthesizes salidroside from tyrosol. These 12 UGTs are known to transfer a glucose group from UDP-glucose to small compounds such as hydroxycinnaamtes like *p*-coumaric acid, caffeic acid, and ferulic acid^20–23^ and monoterpenoids like geraniol and perillyl alcohol^24^. We transformed *E. coli* with each UGT and each transformant was supplemented with tyrosol. *E. coli* strains harboring *AtUGT73C5*, *AtUGT73C6*, or *AtUGT85A1*, yeilded a product that had the same retention time as a standard of salidroside (Fig. 1S). Among these, AtUGT85A1 produced more salidroside than did the others. Therefore, we used AtUGT85A1 for salidroside synthesis.

**Figure 5:**
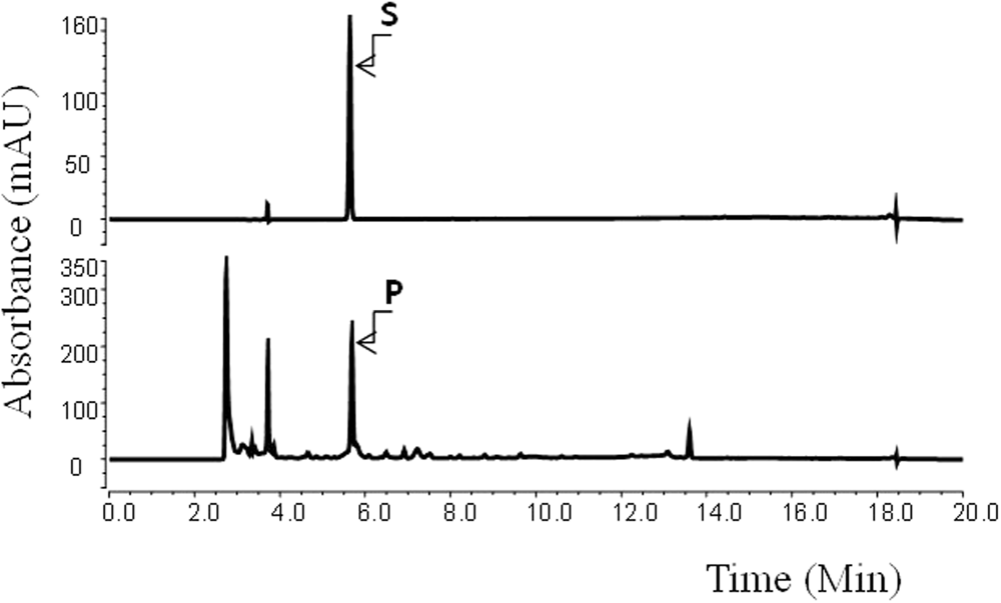
Production of salidroside using strain B-SAL1. S, standard salidroside; P, reaction product.

To synthesize salidroside from glucose, we transformed *E. coli* with both *PcAAS* and *AtUGT84A1*. The resulting transformant, B-SAL1, was used for the production of salidroside. The analysis of the B-SAL1 culture filtrate using HPLC revealed a peak with the same retention time as salidroside (Fig. 5). The tyrosol was not observed, indicating that tyrosol was converted into salidroside as soon as it was produced. The structure of the reaction product was determined to be salidroside by NMR.

**Figure 6:**
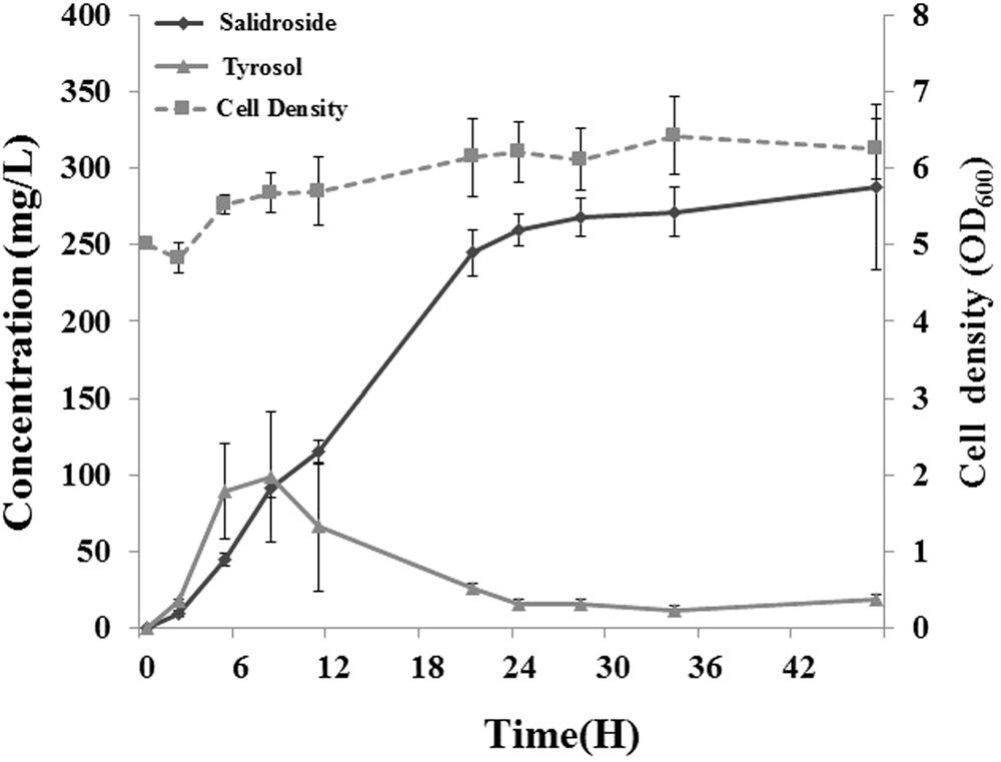
Production of salidroside using strain B-SAL2 after optimization of incubation time and cell concentration.

The synthesis of tyrosol was higher in strain B-TPF. We used B-TPF to synthesize salidroside. The wild type strain (B-SAL1 in Table 1) produced approximately 54.8 mg/L salidroside while the B-TPF strain (B-SAL2 in Table 1) produced 165.8 mg/L, approximately 3-fold more. The optimized reaction time and the initial cell concentration using B-SAL2 were determined to be 25 °C at OD_600_ = 5. Using the optimized incubation temperature and cell concentration, the synthesis of salidroside using B-SAL2 was monitored. Until 8.5 h, both tyrosol and salidroside had accumulated, and after which, the accumulation of saldroside continued to increase until 48 h, at which approximately 287.9 mg/L salidroside was synthesized (Fig. 6). But, tyrosol was converted into salidroside immediately after it was formed. Tyrosol was not observed at the end of the reaction.

### Synthesis of hydroxytyrosol in *E. coli*

Hydroxytyrosol can also be synthesized from tyrosol by hydroxylation. We tested the *HpaBC* gene, encoding 4-hydroxyphenylacetate 3-hydroxylase, from *E. coli* ^25^ and *Sam5* from *Saccharothrix espanaensis* ^26^ in order to convert tyrosol into hydroxytyrosol. HpaBC and Sam5 were used to modify other phenolic compounds such as *p*-coumaric acid, tyrosine, and flavonoids^27–29^. The *HpaBC* or *Sam5* genes were coexpressed with *PcAAS* in *E. coli*, and each transformant was tested for the production of hydroxytyrosol. The transformant harboring *HpaBC* and *PcAAS* produced more hydroxytyrosol than that harboring both *Sam5* and *PcAAS* (data not shown). Therefore, we used *HpaBC* for the synthesis of hydroxytyrosol.

We made two constructs and independently transformed them into *E. coli*, independently. The strain B-HTY1 harbored a single construct containing both *PcAAS* and *HpaBC* and the strain B-HTY2 harbored two separate constructs, one with *PcAAS* and the other with *HpaBC*. We compared the production of hydroxytyrosol. HPLC analysis of culture filtrates from both strains revealed a new peak which had a different retention time with tyrosol. NMR analysis of this peak revealed that the reaction product was hydroxytyrosol. B-HTY1 produced 80.3 mg/L of hydroxytyrol but B-HTY2 produced only 16.6 mg/L, indicating that the strain harboring the single construct produced more tyrosol.

**Figure 7:**
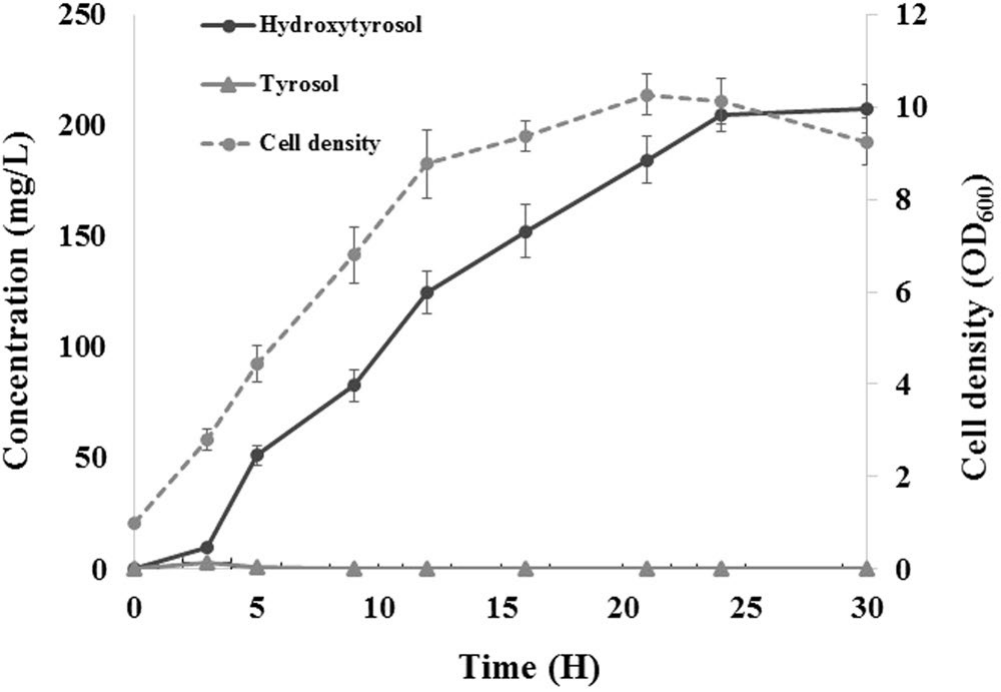
Production of hydroxytyrosol using strain B-THY3 after optimization of incubation time and cell concentration.

We used strain B-TPF to produce hydroxytyrsol because this strain produced more tyrosol than *E. coli* BL21(DE3). The strain B-HTY3 produced 116.7 mg/L tyrosol, more than B-HTY1 (80.3 mg/L) did. Using B-THY3, the optimized initial cell concentration and the incubation temperature were determined to be OD_600_ = 1.0 and 25 °C, respectively. Using the optimized cell concentration and the incubation time, we monitored the production of hydroxytyrosol. As shown in Fig. 7, production of hydroxytyrosol was initially observed after 3 h, and production continued to increase until 30 h, after which 208 mg/L hydroxytyrosol was synthesized. Tyrosol was observed until 5 h at less than 5 mg/L, indicating that tyrosol was converted into hydroxytyrosol as soon as it was produced.

## Discussion

Tyrosol has been synthesized in *E. coli* using *TDC* and *TYO* ^14^ or using *Aro10* from yeast^13^. Hydroxytyrosol was also synthesized using *TH*, *DDC* and *TYO* ^30^ and salidroside was synthesized using *Aro10* and *UGT73B6* ^12^. The protein encoded by *Aro10* uses 4-hydroxypyruvate as a substrate to synthesize 4-HPAA, which undergoes further reduction to make tyrosol. Aro10 does not use tyrosine but 4-hydroxypyruvate as an intermediate for the synthesis of tyrosine. Therefore, they not only used *E. coli* mutants but also overexpressed several genes of the shikimate pathway of *E. coli*. AAS, which exhibits both tyrosine decarboxylase and deaminase activity, converts tyrosine into 4-HPAA and then the endogenous *E. coli* reductase converts it into tyrosol. AAS replaces the activity of both TDC and TYO, and is therefore, an efficient way to synthesize tyrosol from tyrosine in *E. coli*. This is the first report that AAS from plants could synthesize tyrosol as well as hydroxytyrosol and salidroside. We were able to synthesize 531 mg/L tyrosol without overexpressing other genes to increase the content of tyrosine in the cell.

For the synthesis of salidroside, the selection of UGT was critical. We screened 12 UGTs from *A. thaliana* and found AtUGT85A1 as the best UGT for the synthesis of salidroside. AtUGT85A1 is involved in the regulation of the plant hormone trans-zeatin through *O*-glucosylation^31^. It was surprising that AtUGT85A1 also could glucosylate tyrosol to salidroside. Some plant UGTs exhibited substrate promiscuity^32^. Therefore, AtUGT85A1 was capable of utilize diverse sugar acceptors including trans-zeatin, tyrosol, hydroxytyrosol, and 4-hydroxy benzoic acid (data not shown). However, unlike UGT73B6 from *Rhodiola* ^13^, which was previously used for the synthesis of salidroside, AtUGT85A1 exhibited regioselectivity; it did not transfer glucose to the phenolic hydroxyl group to produce icariside. Therefore, the final yield of salidroside was 288 mg/L, which was much higher than that of previous report^12^.

We synthesized tyrosol using *E. coli* harboring *TDC* and *TYO* and compared the final yield to that of *E. coli* harboring *AAS*. The yield of tyrosol using *TDC* and *TYO* (49.2 mg/L) was lower than that using *AAS* (138.9 mg/L). Therefore, we used AAS gene to synthesize tyrosol. When hydroxytyrosol was fed to *E. coli* harboring *AtUGT85A1*, most of hydroxytyrosol was converted into hydroxysalidroside. This indicated that AtUGT85A1 could use hydroxytyrosol as a substrate. However, when hydroxysalidroside was synthesized using B-HTY3 harboring *AtUGT85A1*, only a small amount of hydroxysalidroside was synthesized and more hydroxytyrosol remained. Taken together, it appears that the reaction intermediate(s) inhibits the glycosyltransferase reaction. In particular, the byproduct from the reaction of oxygenase (during salidroside synthesis: TYO; during hyroxysalidroside synthesis: HpaBC) appears to inhibit the UGT acitivity. On the other hand, production of salidroside using Aro10 and UGT73B6 yielded only 56.9 mg/L salidroside while 764.6 mg/L tyrosol remained^13^. Therefore, selection of UGT having high activity as well as the presence of a UGT inhibitor, was critical to the final yield of salidroside.

## Materials and Methods

### Constructs and *E. coli* strains

The *AAS* genes from *A. thaliana*, *P. hybrid*, and *P. crispum* were cloned using reverse transcription polymerase chain reaction (RT-PCR). RNA was isolated from parsley purchased from a local market using the Plant Total RNA Isolation Kit (Qiagen, Velno, Netherlands). cDNA was synthesized as previously described before^33^. Primers for cloning *AAS* were synthesized based on the published parsley AAS sequnence (GenBank accession number: M96070.1): 5′-ATGGATCCGATGGGCTCCATCGATAATCTT-3′ (BamHI site is underlined.) and 5′-ATGCGGCCGCTTATGATAATACTTCCACGA-3′ (NotI site is underlined.); *A. thaniana AAS* gene (AT2G20340.1) 5′-ATGGATCCGATGGAAAATGGAAGCGGGAAG-3′ (BamHI site is underlined.) and 5′-ATGCGGCCGCTTACTTGTGAAGCAAGTAAG-3′ (NotI site is underlined.): *P. hybrid* AAS gene (GenBank accession number: DQ243784.1) 5′-ATGAATTCGATGGATACTATCAAAATCAACCCA-3′ (EcoRI site is underlined.) and 5′-ATGCGGCCGCCTACGCATTCAGCATCATAGTT-3′ (NotI site is underlined.). Each *AAS* gene was subcloned into the corresponding sites of the pCDF-Duet1 vector.

Tyrosine decarboxylase (TDC from *Papaver somniferum*; GenBank U08598.1), and tyrosine oxidase (TYO from *Micrococcus luteus*; GenBank AB010716.1) were synthesized after codon optimization using the published nucleotide sequences (Figs 2S and 3S). TDC was subcloned into BamHI/HindIII site of pCDF-Duet1 vector, and the resulting construct was pC-TDC. TYO was introduced into the second cloning site (NdeI/XhoI) of pC-TDC and the resulting constructs was called pC-TDC-TYO.

Twelve UGTs (AtUGT71C1 [At2g29750], AtUGT71C2 [At2g29740], AtUGT72B1 [At4g01070], AtUGT72E2 [At5g66690], AtUGT73C1[At2g36750], AtUGT73C3 [At2g36780], AtUGT73C5 [At2g36800], AtUGT73C6 [At2g36790], AtUGT76D1 [At2g26480], AtUGT76E2 [At5g59590], AtUGT76E12 [At3g46660], AtUGT85A1 [At1g22400]) from *A. thaliana* were subcloned into the pGEX 5X-3 vector.

The list of constructs and *E. coli* strains used in this study can be found in Table 1.

### Production of tyrosol, hydroxytyrosol, and salidroside in *E. coli*

For the synthesis of tyrosol, hydroxytyrosol, and salidroside, *E. coli* was grown in LB medium containing 50 μg/mL appropriate antibiotics at 37 °C for 18 hr. The seed culture was inoculated into a fresh LB medium containing antibiotics and incubated at 37 °C until OD_600_ = 1.0. Cells were harvested by centrifugation, washed once with M9 medium. For the initial screening of AAS gene, the cell was resuspended with M9 containing 2% glucose, 50 μg/mL antibiotics, 1 mM IPTG, and 100 μM. For the synthesis of tyrosol, hydroxytyrosol, and salidroside, cells were resuspended with M9 containing 2% glucose, 50 μg/mL antibiotics, 1 mM IPTG, and 0.1% yeast extract. The cell density was adjusted to OD_600_ = 1.0. The resulting culture was incubated at 30 °C for 24 h. To detect tyrosol and hydroxytyrosol production, the culture was extracted with ethylacetate and the organic layer was collected after centrifugation and evaporated to dryness. The remaining reaction product was dissolved with dimethyl sulfoxide (DMSO) and analyzed by Thermos high performance liquid chromatography (HPLC). To examine the production of salidroside, the culture was boiled for 3 min and then centrifuged. The supernatant was filtered using 0.45 μm syringe filter (Millipore, Billerica, MA, USA) and analyzed by HPLC.

To analyze the formation of tyrosol, hydroxytyrosol, and salidroside by HPLC, the mobile phase was composed of water (solution A) and acetonitrile (solution B), which were combined with 0.1% formic acid. The elution program was as follows: the proportion of solution B was gradually increased from 10% to 40% over 8 min, increased to 90% over 4 min, and then maintained over 3 min. Finally, the proportion of solution B was rapidly decreased to 10% and maintained at that composition for 5 min. The flow rate wass 1 ml/min.

The structure of reaction product was determined using nuclear magnetic resonance (NMR) spectroscopy^34, 35^ (NMR spectra was provided in the Figs 4S, 5S, and 6S). The NMR data were as follows; Tyrosol, 1 H NMR (400 MHz, CDCl3): δ 7.10 (d, J = 8.3 Hz, 2 H), 6.78 (d, J = 8.3 Hz, 2 H), 3.83 (t, J = 6.5 Hz, 2 H), 2.80 (t, J = 6.5 Hz, 2 H).

Salidroside, ^1^H NMR (400 MHz, DMSO-*d*_6_) *δ* 7.04 (d, *J* = 8.3 Hz, 2 H), 6.67 (d, *J* = 8.3 Hz, 2 H), 4.17 (d, *J* = 8.5 Hz, 1 H), 3.87 (dd, *J* = 16.0, 8.6 Hz, 1 H), 3.67 (d, *J* = 11.4 Hz, 1 H), 3.56 (dd, *J* = 16.0, 8.8 Hz, 1 H), 3.44 (dd, *J* = 11.4, 5.4 Hz, 1 H), 3.15 (t, *J* = 8.5 Hz, 1 H), 3.07 (m, 1 H), 3.06 (d, *J* = 8.5 Hz, 1 H), 2.96 (t, *J* = 8.5 Hz, 1 H), 2.73 (m, 2 H); ^13^C NMR (100 MHz, DMSO-*d*_6_) *δ* 155.6, 129.7, 128.6, 115.0, 102.8, 76.8, 76.7, 73.4, 70.0, 69.9, 61.0, 34.8.

Hydroxytyrosol, ^1^H NMR (400 MHz, MeOD): *δ* 6.68 (d, *J* = 8.0 Hz, 1 H), 6.66 (d, *J* = 1.9 Hz, 1 H), 6.53 (dd, *J* = 8.0, 2.0 Hz, 1 H), 3.68 (t, *J* = 7.2 Hz, 2 H), 2.66 (t, *J* = 7.2 Hz, 2 H).

## Electronic supplementary material

Supplementary Info
