# Supplementary material for: Production of three phenylethanoids, tyrosol, hydroxytyrosol, and salidroside, using plant genes expressing in Escherichia coli
Source: Sci Rep. 2017 May 31;7:2578. doi: 10.1038/s41598-017-02042-2 (PMC5451403; doi:10.1038/s41598-017-02042-2)
Supplement: Supplementary file 1 — Supplementary Info [file 41598_2017_2042_MOESM1_ESM.pdf]

Supplementary information

Production of three phenylethanoids, tyrosol, hydroxytyrosol, and salidroside, using plant genes expressing in *Escherichia coli*

Daeun Chung [1], So Yeon Kim[1], Joong-Hoon Ahn*

Department of Integrative Bioscience and Biotechnology, Bio/Molecular Informatics Center, Konkuk University, Seoul 05029, Republic of Korea

*Corresponding author

Phone: +82-2-45-3764; Fax: +82-2-3437-6106

E-mail: jhahn@konkuk.ac.kr

[1]These authors contributed equally to this work.

Supplementary Information

Figure 1S. Screening of 12 UGTs for the synthesis of salidroside from tyrosol.

Figure 2S. Codon optimized nucleotide sequence of *TYO* from *Micrococcus luteus.*

Figure 3S. Codon optimized nucleotide sequence of TDC from *Papaver somniferum.*

Figure 4S. NMR spectra of tyrosol.

Figure 5S. NMR spectra of hydroxytyrosol.

Figure 6S. NMR spectra of synthesized salidroside. a, ^{1}H-NMR; b, ^{13}C-NMR; c, DEPT-135; d, COSY; e, HMBC.

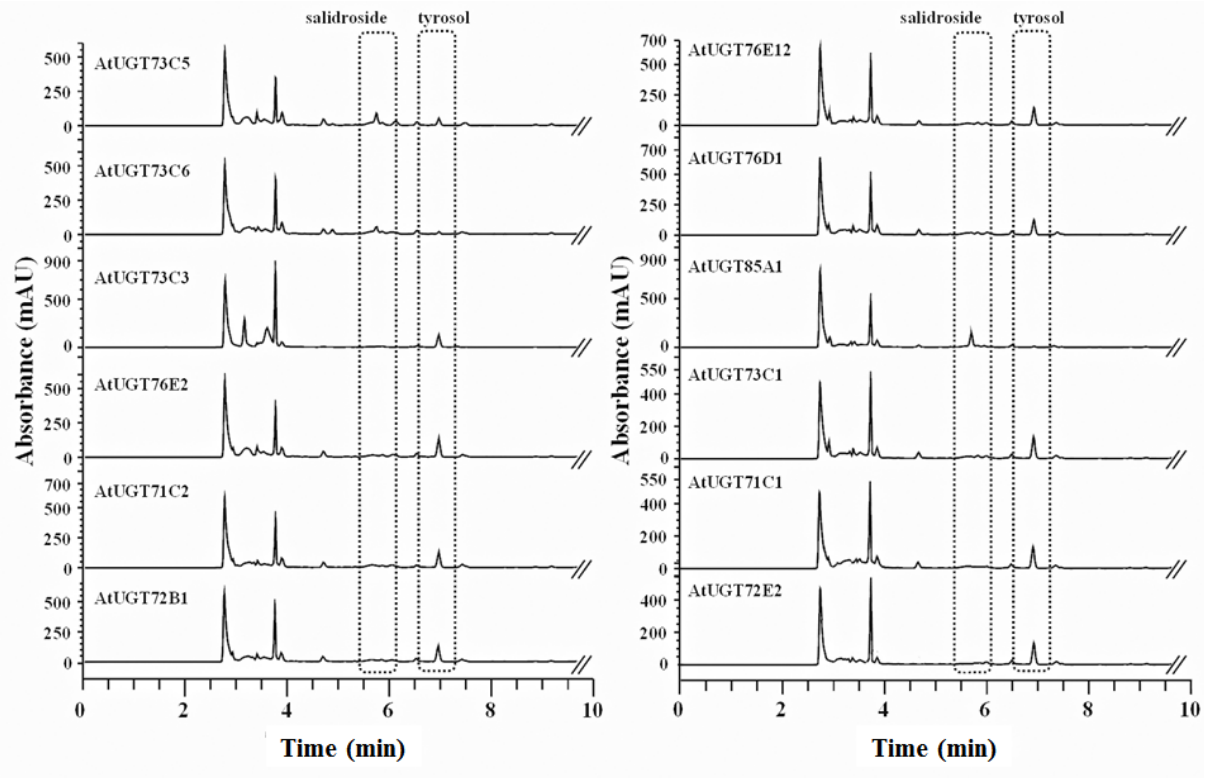

Figure 1S. Screening of 12 UGTs for the synthesis of salidroside from tyrosol.

ATGTCTAATCCGCATGTTGTGATCGTCGGTGCCGGTTTTGCCGGCCTGGTGGCTGCCCGTGAACTGCAAATGGCC
GGCGTGGATGTGGAGATCGTGGAGGCTCGCGACCGCGTAGGAGGTCGCGCCTGGACCGAAGAAAGGATGGGCCGT
CCACTGGAACTGGGTGCCACGTGGGTGCATTGGATGCAGCCGCATGTGTGGAGTGAGATCACTCGCTACGACCAG
TCAATTTATCCCAGCCCTTTTTGCGACGACGCATACTGGATCACTGGTGGCCGGGTTGAACACGGCACCGAAGCG
GACTTGGATGCTGCTCTGGCACGCCCGATGGCCAAAATCTTTGAAGATTCGCGTGAGTTCTTCCCGTATCCTTAT
GAGCCACTGCACGTTCTGGATGAAAGTAGTGGCAGCACACCCGAGCTGCGGGAAAGGTTTAGAGCTGCGGACCAA
GGCAGTGTGCTGGATTGTCTCAAAGGCGGCGACTTTACCCAGGAGGAGAGAGATTTGTGCGACGCGTATTGGTCC
GCTGCTTATATAGGGGACCCGCATCAAGGTTCACCGTTAATGGCTAAACAGTGGGCGGCCCTGTCCGATCACCGG
TTGAGCCTGGTGGACGAGCAGACCCTGCGCTTCAAGTTAACCCACGGCATGCGTGGACTGTATGAGAATATAGCC
GCGGATCTGCGCTGCCCTATTCGCTTAAACACCCCGGTCACGGCGGTCGATCATCGCTCCGACGGCGCCACAGTT
ACCCTTGGAACTGGTGAAAAAATTTCATGTGACTCAGTGATAGTTACAGTTCCGGTGGGGGCGCTACCAACCATT
GAGTTTACTCCGGGGCTGCCCTCGGGGATGCGCACCGTGATTGATCAGAGATGGAACTCTACTGGTTGTAAAATA
TGGGTCAAAGTCAAAGGTCATCATTCCATTCTGGGTTACGCCCCCACGCCTCATAAGGCCGCCGTTTTTCGCAGC
GAGTTCTTCATGGATGACGACACAACCATTTGCGTGGGCTTTGGTTCTCACCATGATGCCGTGGATCTTACGGAC
CCACGGGACGCCCAGGCAATCGTGGACCAGTGGCGTCCAGACTTGGAGGTGGTGGACTGTACTGGCCATGATTGG
GTGGCGGACAGGTGGAGCGGTCAAGCGTGGGCTACCCTGCGATCTGGGCAATTCACCAATGGCTGGCACCATTTT
CGATCTACAGATTCGCGTCTTCGCTTCGCCGGGGCGGATTGGGCGCGTGGTTGGCGCGGAGTGGTTGTGGACGGT
GCCATCGAGACGGGTCTGAGTACGGCCCGCGACGTCCTCCGAGACATCCGCGCATAA

Figure 2S. Codon optimized nucleotide sequence of *TYO* from *Micrococcus luteus*.

ATGGGTTCTCTTAACACTGAGGATGTTCTTGAAAACAGTTCGGCCTTCGGCGTAACAAACCCATTAGACCCAGAA
GAATTTCGCAGACAGGGTCACATGATAATCGACTTCTTAGCTGATTATTATCGCGATGTCGAAAAATATCCAGTT
CGGAGTCAGGTAGAACCTGGTTATCTACGTAAACGCCTCCCTGAGACCGCTCCGTACAATCCAGAATCGATAGAA
ACGATTCTTCAAGATGTGACTACTGAAATTATCCCAGGATTAACCCATTGGCAGAGTCCGAATTACTATGCTTAT
TTCCCTTCGAGCGGTTCCGTGGCCGGGTTCCTCGGCGAAATGCTTAGTACTGGCTTTAATGTTGTTGGTTTTAAC
TGGATGTCTTCACCTGCTGCGACTGAATTAGAAAGTGTCGTTATGGATTGGTTCGGGAAAATGCTTAACCTTCCA
GAATCATTCTTGTTCAGCGGTTCTGGCGGTGGCGTTTTGCAAGGAACCTCCTGTGAGGCAATTTTATGCACATTA
ACAGCGGCGAGGGATAGAAAGCTCAACAAAATAGGTCGTGAACATATCGGAAGGCTCGTTGTGTATGGATCTGAT
CAAACGCACTGTGCACTACAAAAAGCAGCCCAGGTCGCAGGCATTAACCCTAAGAACTTCCGTGCGATTAAAACG
TTTAAAGAAAACTCGTTTGGACTGTCAGCGGCTACCCTGCGGGAAGTAATTCTTGAAGACATTGAAGCCGGGCTC
ATCCCGCTTTTCGTATGTCCCACGGTCGGAACTACATCATCTACCGCGGTGGACCCGATCTCTCCGATCTGTGAA
GTGGCAAAAGAGTATGAGATGTGGGTTCATGTAGACGCAGCGTATGCTGGCTCGGCGTGTATCTGCCCTGAGTTT
CGCCACTTCATCGACGGTGTTGAGGAAGCTGATTCATTCAGTCTCAATGCACATAAATGGTTTTTTACAACCTTG
GATTGCTGCTGCTTATGGGTCAAAGATCCATCAGCCCTTGTTAAAGCCCTTTCCACAAATCCCGAATACTTGCGT
AATAAAGCCACGGAGAGCCGGCAGGTTGTTGACTATAAAGACTGGCAGATCGCGCTCAGTCGCCGATTTCGGTCC
TTGAAACTTTGGATGGTCTTACGTAGCTATGGCGTAACGAATTTGAGAAACTTTCTGCGCTCCCACGTTAAAATG
GCTAAAACATTCGAGGGTCTTATTTGTATGGATGGGCGTTTTGAGATTACCGTGCCGCGGACTTTTGCGATGGTC
TGTTTTCGACTTCTCCCGCCGAAGACCATAAAGGTGTACGACAATGGTGTTCACCAGAATGGAAATGGGGTAGTT
CCCCTACGTGATGAAAATGAAAATTTAGTGCTGGCCAATAAGTTAAATCAGGTTTATTTGGAGACAGTCAATGCC
ACGGGCTCTGTTTACATGACTCATGCCGTCGTTGGCGGTGTCTACATGATTCGGTTTGCAGTGGGTTCAACCCTC
ACTGAGGAACGGCATGTTATATATGCATGGAAGATTTTACAAGAGCATGCAGACCTGATTCTTGGTAAGTTCAGT
GAAGCTGATTTTTCAAGTTAA

Figure 3S. Codon optimized nucleotide sequence of TDC from *Papaver somniferum.*

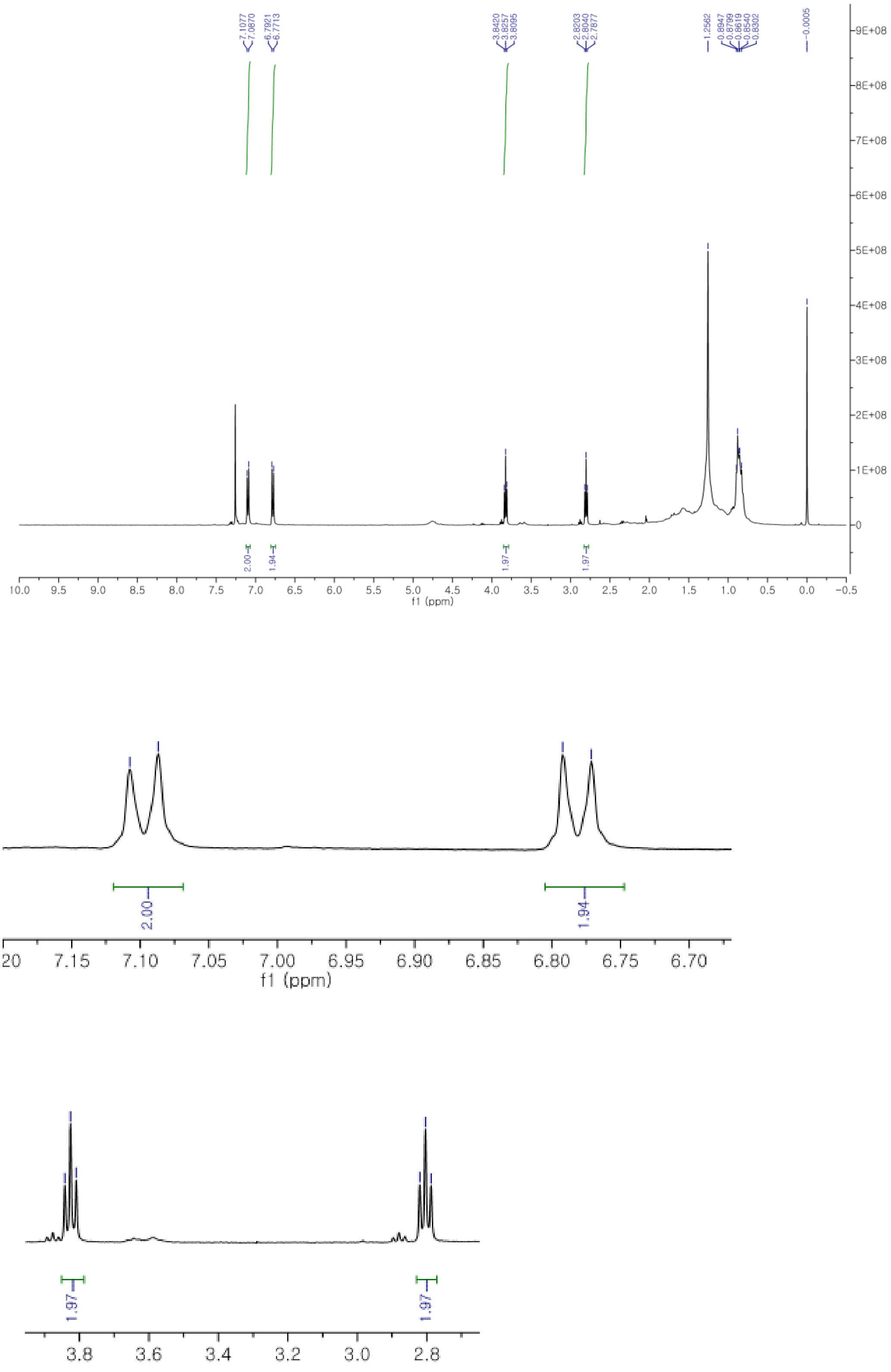

Figure 4S. NMR spectra of tyrosol.

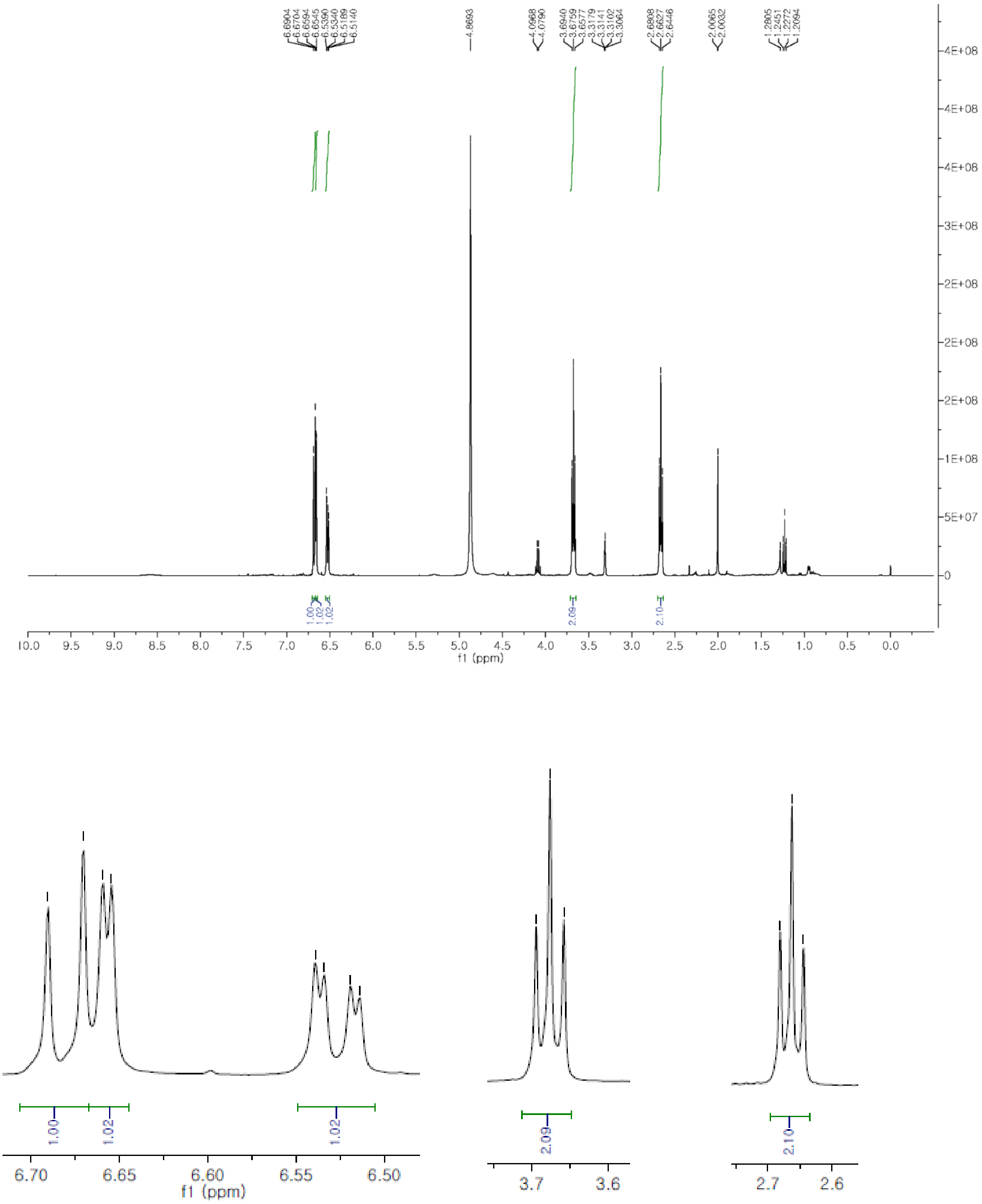

Figure 5S. NMR spectra of hydroxytyrosol.

(a)

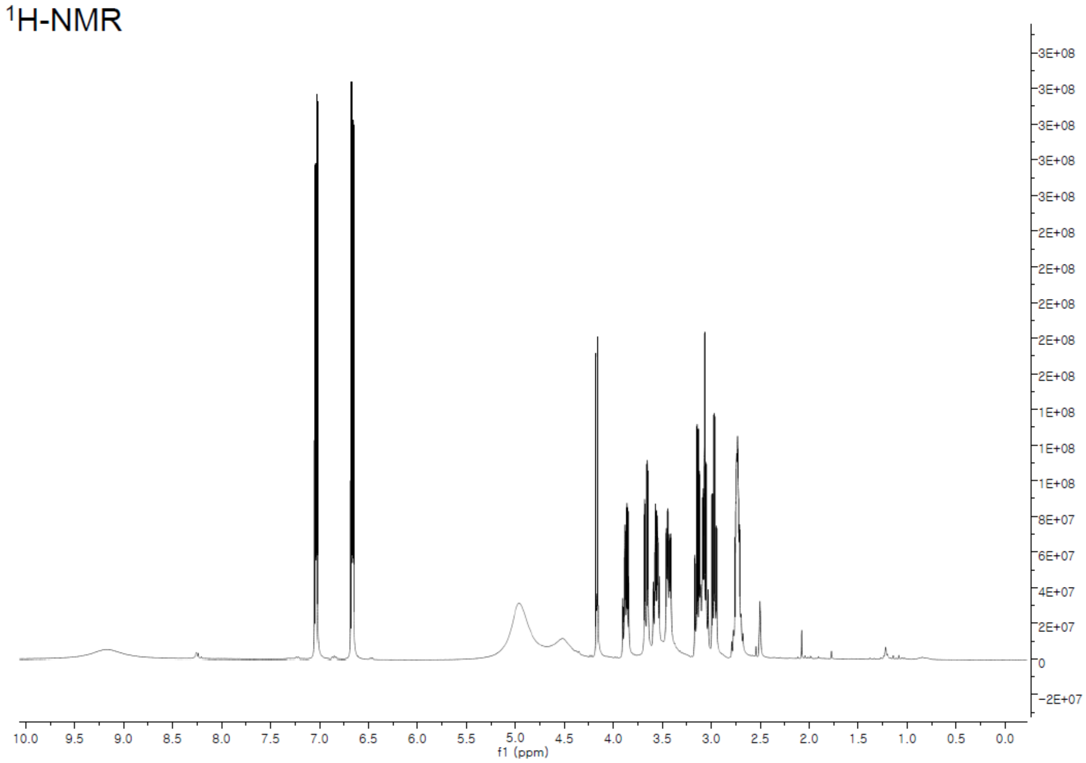

(b)

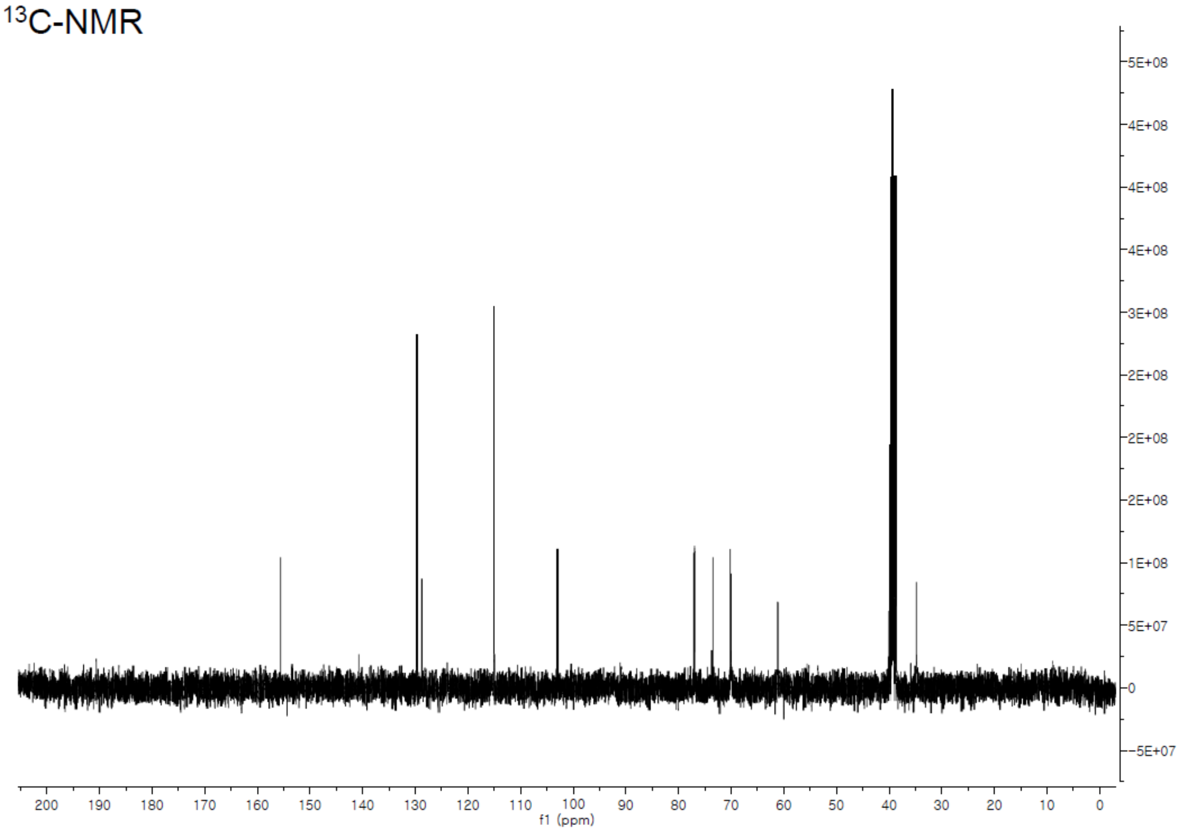

^{13}C-NMR
5E+08
4E+08
4E+08
4E+08
3E+08
2E+08
2E+08
2E+08
1E+08
5E+07
0
-5E+07
200
190
180
170
160
150
140
130
120
110
100
90
80
70
60
50
40
30
20
10
0
f1 (ppm)

(c)

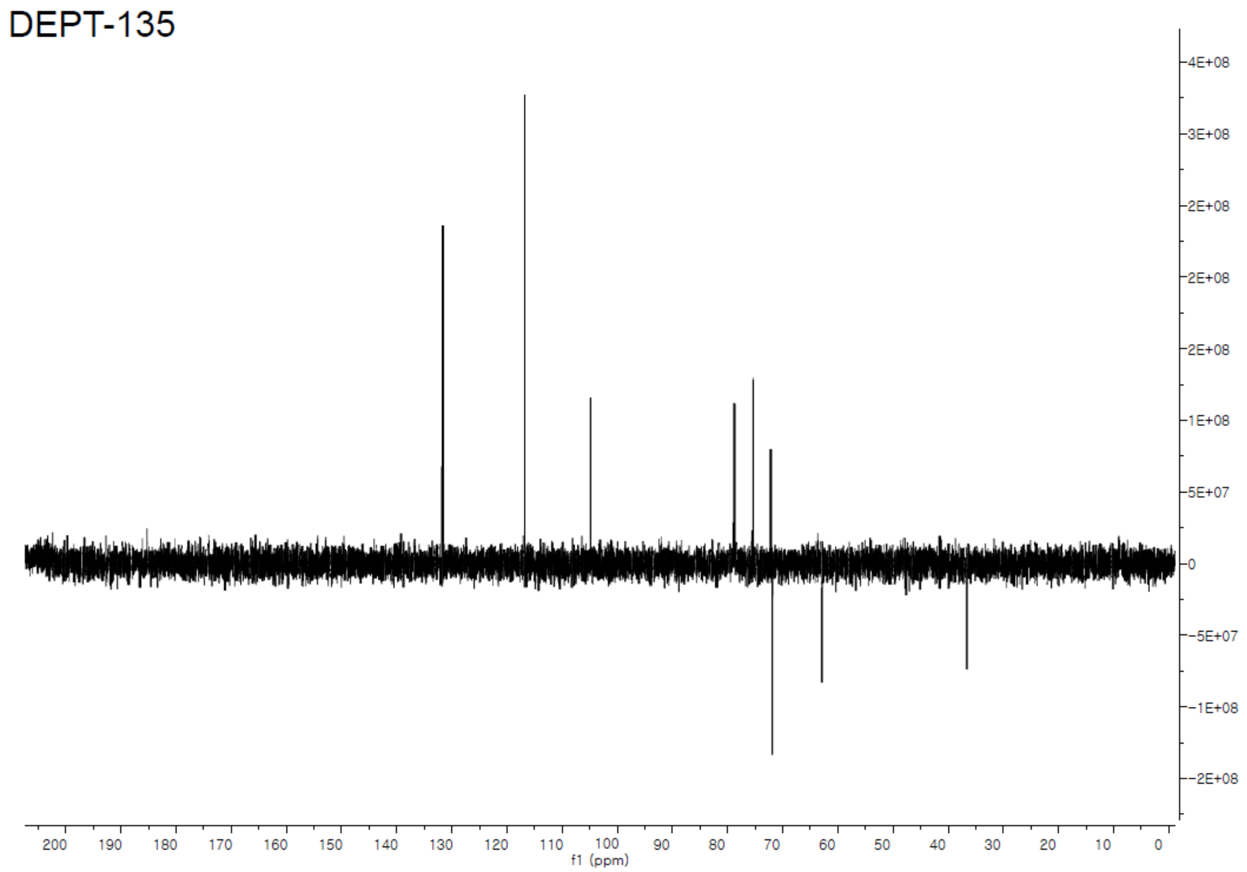
DEPT-135
4E+08
3E+08
2E+08
2E+08
2E+08
1E+08
5E+07
0
−5E+07
−1E+08
−2E+08
200
190
180
170
160
150
140
130
120
110
100
90
80
70
60
50
40
30
20
10
0
f1 (ppm)

(d)

COSY

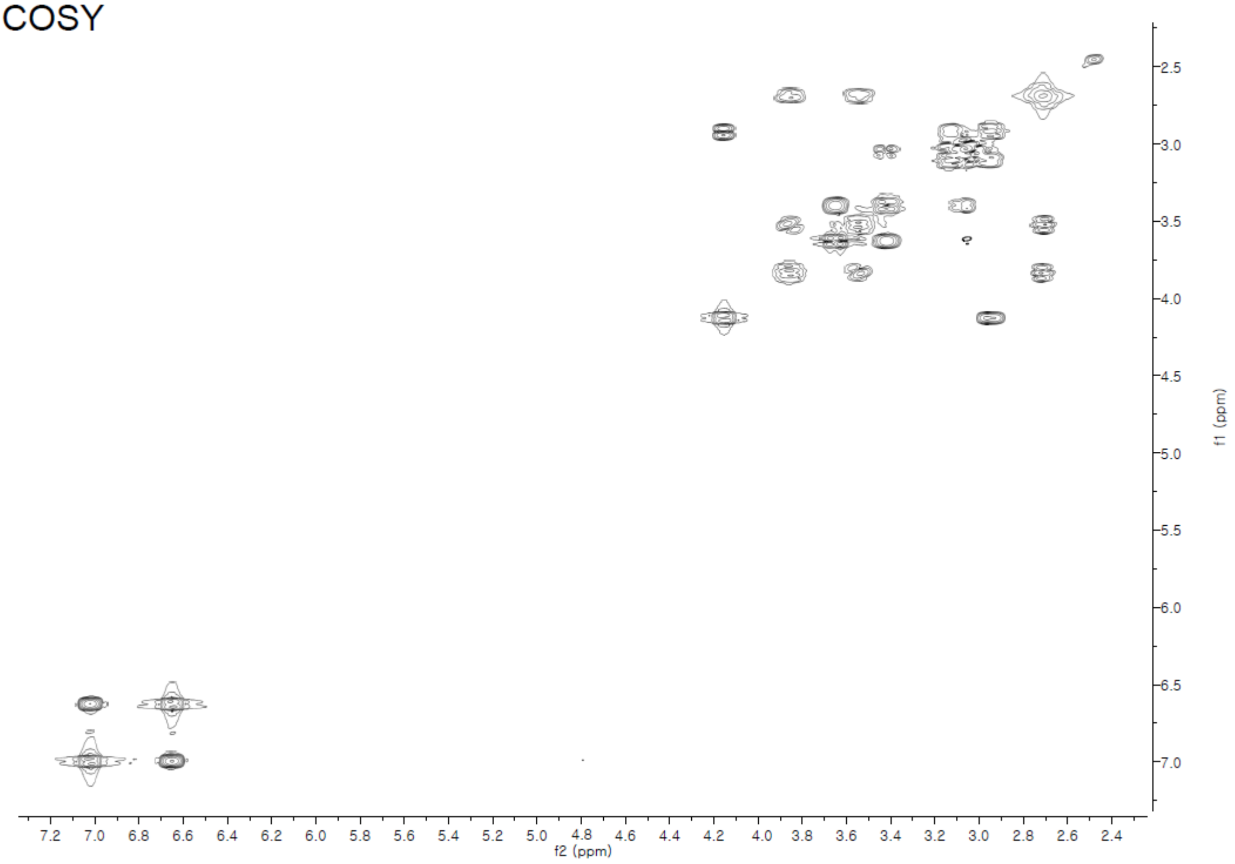

f1 (ppm)
2.5
3.0
3.5
4.0
4.5
5.0
5.5
6.0
6.5
7.0
7.2 7.0 6.8 6.6 6.4 6.2 6.0 5.8 5.6 5.4 5.2 5.0 4.8 4.6 4.4 4.2 4.0 3.8 3.6 3.4 3.2 3.0 2.8 2.6 2.4
f2 (ppm)

(e)

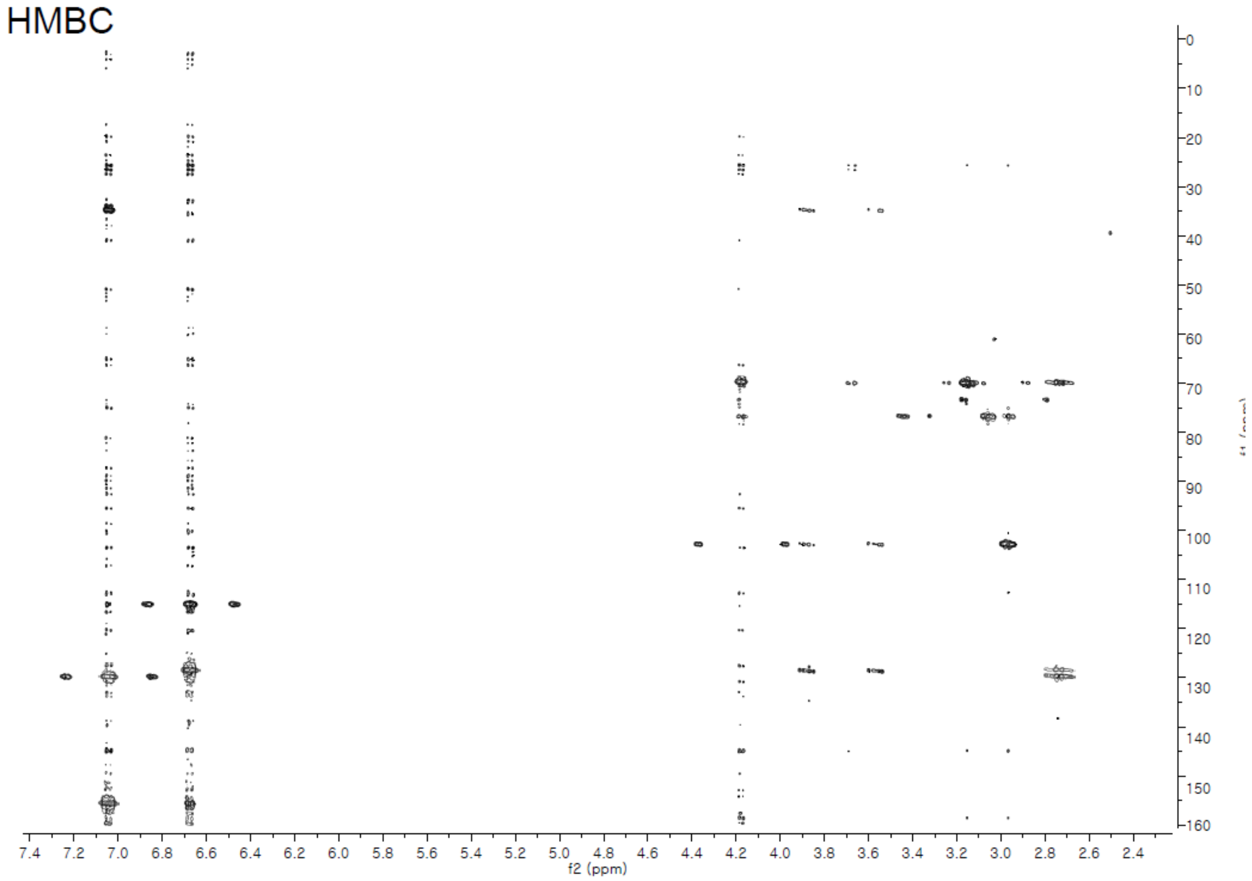

Figure 6S. NMR spectra of synthesized salidroside. a, ^{1}H-NMR; b, ^{13}C-NMR; c, DEPT-135; d, COSY; e, HMBC.
